# Toward a Multiscale Description of Microvascular Flow Regulation: O_2_-Dependent Release of ATP from Human Erythrocytes and the Distribution of ATP in Capillary Networks

**DOI:** 10.3389/fphys.2012.00246

**Published:** 2012-07-16

**Authors:** Daniel Goldman, Graham M. Fraser, Christopher G. Ellis, Randy S. Sprague, Mary L. Ellsworth, Alan H. Stephenson

**Affiliations:** ^1^Department of Medical Biophysics, University of Western OntarioLondon, ON, Canada; ^2^Department of Pharmacological and Physiological Science, Saint Louis University School of MedicineSt. Louis, MO, USA

**Keywords:** oxygen supply regulation, signal pathway modeling, ATP transport model, O_2_ transport model

## Abstract

Integration of the numerous mechanisms that have been suggested to contribute to optimization of O_2_ supply to meet O_2_ need in skeletal muscle requires a systems biology approach which permits quantification of these physiological processes over a wide range of length scales. Here we describe two individual computational models based on *in vivo* and *in vitro* studies which, when incorporated into a single robust multiscale model, will provide information on the role of erythrocyte-released ATP in perfusion distribution in skeletal muscle under both physiological and pathophysiological conditions. Healthy human erythrocytes exposed to low O_2_ tension release ATP via a well characterized signaling pathway requiring activation of the G-protein, Gi, and adenylyl cyclase leading to increases in cAMP. This cAMP then activates PKA and subsequently CFTR culminating in ATP release via pannexin 1. A critical control point in this pathway is the level of cAMP which is regulated by pathway-specific phosphodiesterases. Using time constants (~100 ms) that are consistent with measured erythrocyte ATP release, we have constructed a dynamic model of this pathway. The model predicts levels of ATP release consistent with measurements obtained over a wide range of hemoglobin O_2_ saturations (sO_2_). The model further predicts how insulin, at concentrations found in pre-diabetes, enhances the activity of PDE3 and reduces intracellular cAMP levels leading to decreased low O_2_-induced ATP release from erythrocytes. The second model, which couples O_2_ and ATP transport in capillary networks, shows how intravascular ATP and the resulting conducted vasodilation are affected by local sO_2_, convection and ATP degradation. This model also predicts network-level effects of decreased ATP release resulting from elevated insulin levels. Taken together, these models lay the groundwork for investigating the systems biology of the regulation of microvascular perfusion distribution by erythrocyte-derived ATP.

## Introduction

The regulation of blood flow involves interplay among numerous mechanisms including the tissue specific microvascular architecture, wall shear stress and pressure (myogenic tone), and the activity of the sympathetic nervous system. Although each of these clearly contributes to total microvascular perfusion, these factors alone are insufficient to regulate dynamically the precise distribution of perfusion to meet local tissue oxygen (O_2_) need. Such a system requires a mechanism by which the need is detected, quantified, and coupled to a mechanism for the alteration of O_2_ delivery. A number of theories have been proposed by which blood flow can be increased in response to decreases in tissue oxygen tension including the arterioles themselves being sensitive to low O_2_ levels (Pittman and Duling, 1973; Duling, 1974; Jackson, 1987) the release of vasodilatory metabolites within the tissues or vessels (Hester, 1993), and more recently the release of nitric oxide (Jia et al., 1996) and/or nitrite (Gladwin et al., 2004) from erythrocytes. Although each may play a role, none provides the sensitivity and rapid time course necessary for the precise matching of oxygen supply with need.

One mechanism which has been the subject of significant interest in recent years involves the regulated release of ATP (adenosine triphosphate) from erythrocytes in response to a decrease in hemoglobin oxygen saturation (sO_2_; Ellsworth et al., 1995, 2009; Jagger et al., 2001) as would result from their exposure to a reduced oxygen tension environment. The ATP released would bind to endothelial purinergic receptors inducing vasodilation via the synthesis and release of endothelium-derived relaxing factors. Experimentally, studies have established that infusion of ATP into hamster skeletal muscle arterioles and venules, at concentrations observed *in vivo* (Gonzalez-Alonso et al., 2002), induces a vasodilation that is conducted upstream to feed arterioles resulting in increased perfusion (McCullough et al., 1997; Collins et al., 1998). Such a mechanism would permit the erythrocyte, via a local release of ATP, to evoke an increase in O_2_ supply to discrete regions of the microvasculature enabling the dynamic changes in O_2_ delivery needed to meet changing local tissue oxygen needs. For this mechanism to be effective, the amount of ATP released from erythrocytes needs to be directly related to the extent of hemoglobin O_2_ desaturation (or decrease in sO_2_) that occurs when erythrocytes are exposed to low O_2_ tension (or partial pressure, PO_2_; Jagger et al., 2001).

Several recent reviews (Ellsworth, 2000, 2004; Gonzalez-Alonso, 2008; Ellsworth et al., 2009; Sprague et al., 2011) and previous theoretical models (Arciero et al., 2008; Sprague et al., 2010) have evaluated the impact of erythrocyte-released ATP on microvascular flow regulation. However, a full understanding of the effect of erythrocyte-derived ATP on the regulation of O_2_ delivery requires quantification of the interacting physiological processes over a wide range of physical length scales. To accomplish this necessitates the incorporation of several individual experiment-based computational models into a novel multiscale model. Two critical components of such a dynamic model are delineated here.

**Figure 1:**
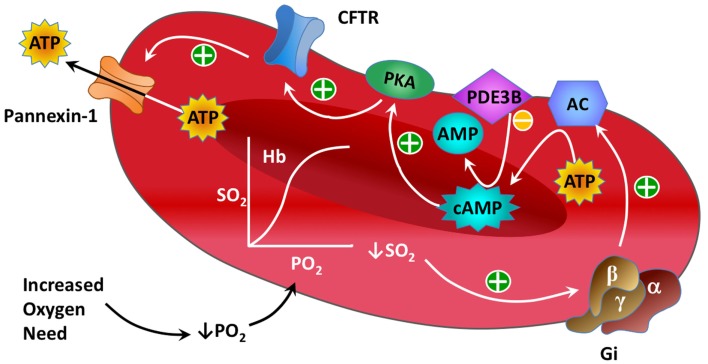
**Illustration showing known components of the oxygen-dependent erythrocyte ATP release pathway**.

Significant progress has been made in defining the components of a signaling pathway for ATP release from erythrocytes under conditions of low O_2_ tension (Ellsworth et al., 2009). Important elements of this pathway (see Figure 1) include activation of the heterotrimeric G-protein Gi (Sprague et al., 2002; Olearczyk et al., 2004a,b) and, subsequently, the activation of adenylyl cyclase (AC) resulting in increases in intracellular cyclic adenosine monophosphate (cAMP; Sprague et al., 2002, 2005, 2006). This results in activation of protein kinase A (PKA; Sprague et al., 2001) and the cystic fibrosis transmembrane conductance regulator (CFTR; Sprague et al., 1998). The final conduit for ATP release in response to this stimulus has been determined to be pannexin 1 (Locovei et al., 2006; Sridharan et al., 2010).

Recent experimental studies demonstrate that insulin, at concentrations observed in humans with pre-diabetes and used to treat individuals with diabetes mellitus type 2 (type 2 diabetes), inhibits low O_2_ tension-induced ATP release from human erythrocytes (Hanson et al., 2009). Importantly, animal studies suggest that this defect contributes to the impaired tissue oxygenation in pre-diabetes (Ellis et al., 2010). A critical control point in the low O_2_ signaling pathway for regulated ATP release is the concentration of cAMP which is determined by a balance between cAMP synthesis by adenylyl cyclase and its hydrolysis by phosphodiesterases (PDEs). Insulin has been shown to increase hydrolysis of cAMP via the enhancement of PDE3 activity (Hanson et al., 2010).

Although signaling pathways similar to the one responsible for ATP release from erythrocytes have been described previously in other cells (Cazzaniga et al., 2008; Williamson et al., 2009), no quantitative approach has integrated the signaling components of low O_2_ tension-induced ATP release from erythrocytes into a unified mathematical framework that would permit the comprehensive study of its regulation. Here we present a single compartment kinetic model of the low O_2_ tension-induced ATP release pathway in human erythrocytes that incorporates parameters consistent with experimentally measured ATP release in response to this stimulus in the absence (Sprague and Ellsworth, 2012) and presence (Hanson et al., 2009) of insulin. This model, based on a previously described deterministic model of a G-protein coupled cAMP pathway (Williamson et al., 2009), incorporates interactions among individual cellular components based on our current understanding of the signaling pathway for low O_2_ tension-induced ATP release. Our approach involves the use of data obtained from experimental studies including those defining PDE3-mediated cAMP hydrolysis as a critical control point for the regulation of low O_2_-induced ATP release from human erythrocytes (Hanson et al., 2010).

In addition to our model of the intracellular ATP release pathway, we utilized *in vivo* data obtained from rat skeletal muscle to construct a realistic model of blood flow, O_2_ transport, and ATP transport at the capillary network level to investigate the impact of low O_2_-induced ATP release from erythrocytes on the regulation of perfusion distribution in skeletal muscle under physiological conditions and when plasma insulin is increased. Our model of capillary network ATP transport, although used to obtain steady-state results in the present work, is novel in that it is time-dependent and hence permits simulation of the dynamics of this process. This model will be crucial in future studies of microvascular flow regulation, which is an inherently dynamic physiological process (e.g., due to temporal variations in both local blood flow and O_2_ consumption rate), and will allow us to include the ATP release dynamics from our pathway model into a larger-scale model of flow regulation in complete networks containing capillaries, arterioles, and venules.

Our underlying hypothesis is that the O_2_-dependent release of ATP from erythrocytes is a key mechanism for the dynamic regulation of the distribution of microvascular perfusion to meet local tissue O_2_ needs in skeletal muscle. The long-term goal is to utilize a combination of computational models and experimental studies to ascertain how and under what conditions ATP release from erythrocytes contributes to appropriate O_2_ delivery. Furthermore, the use of computational models provides a mechanism by which predictions of impaired ATP release based on known defects associated with certain disease states, and the potential effectiveness of pharmacological interventions to rescue the defect, can be evaluated. Experimental data supporting the stated hypothesis have been reported previously (Collins et al., 1998; Dietrich et al., 2000; Sprague et al., 2009) and were used as a basis for constructing the models. The two components described here complement models of 3D blood-tissue O_2_ transport and two-phase blood flow presented previously and will become an important part of a multiscale simulation required to characterize flow regulation based on ATP release from erythrocytes.

## Materials and Methods

### Simplified model of O_2_-dependent Gi-activated cAMP pathway

As described above, the basic components of heterotrimeric G-protein (GP)-activated signaling pathways involving cAMP are well-known in many cell types including the erythrocyte. To begin modeling the key components of the O_2_-dependent erythrocyte ATP release pathway (Figure 1), we modified a simple model of a GP-activated cAMP pathway from the literature (Williamson et al., 2009). Although the exact mechanism that couples a decrease in hemoglobin saturation with GP activation has not been fully elucidated, several studies have linked mechanical force with activation of Gi (Li and Xu, 2000; Wan et al., 2008; Forsyth et al., 2011). Our model requires that the desaturation of oxyhemoglobin induces activation of Gi, identified here as the activated form of GP (GPa). When erythrocyte Gi dissociates, the βγ subunit stimulates production of cAMP (via adenylyl cyclase, AC; Sprague et al., 2002, 2005, 2006) leading to activation of protein kinase A (PKAi → PKAa; Sprague et al., 2001, 2006). The kinetic equations adapted for the activation of PKA in the present model are:

(1) $$\frac{d\left[\text{GPa}\right]}{dt}=k_{\text{GPf}}\left[\text{GPi}\right]{\left[\text{tHb}\right]}^{\alpha }-k_{\text{GPr}}\left[\text{GPa}\right]$$

(2) $$\frac{d[c\text{AMP}]}{dt}-\frac{{\text{AC}}_{base}+k_{\text{cAM}Pf}[\text{GP}a]}{1+k_{\text{cAMPi}}[\text{PKAa}]}-\frac{\nu_{\text{PDE3}}[\text{PKA}a][c\text{AMP}]}{K_{\text{PDE}3}+[c\text{AMP}]}$$

(3) $$\frac{d[\text{PKAa}]}{2}=k_{\text{PKAf}}[\text{PKAi}][\text{cAMP}]-k_{\text{PKAr}}[\text{PKAa}]$$

Equation 1 above describes GP activation resulting from changes in oxyhemoglobin saturation where [tHb] is the fraction of desaturated Hb (in the tense or “t” state; [tHb] = 1 − sO_2_) and the exponent *α* is used to modulate the relationship between [tHb] and GPa. Equation 2 describes cAMP production and degradation, where AC_base_ represents the baseline rate of cAMP production (in the absence of GP activation) and the *v*_PDE3_ term represents degradation of cAMP by the phosphodiesterase PDE3, a PDE shown to regulate cAMP concentrations in the erythrocyte O_2_-dependent ATP release pathway (Adderley et al., 2010). Here, *v*_PDE3 _= v_0_*PDE3*_rel_* where *v_0_* is a baseline rate of cAMP degradation and PDE3*_rel_* is the relative amount of PDE3 activity (assumed to be one under normal baseline conditions). The PKAa terms on the right-hand side represent negative feedback to either inhibit cAMP production (Sobolewski et al., 2004) or enhance cAMP degradation (Murthy et al., 2002). Eq. 3 represents direct activation of PKA by cAMP. In all our kinetic equations, the subscript “f” indicates the forward rate constant (e.g., *k*_PKAf_) governing production of the species of interest, while the subscript “r” indicates the reverse rate constant (e.g., k*_PKAr_*) governing degradation.

### PKA/CFTR-activated ATP release

To link the PKA activation as described in Eq. 3 to the release of ATP, two kinetic equations are employed that describe the other known regulatory steps in the process:

(4) $$\frac{d[\text{CFTRa}]}{dt}=k_{\text{CFTRf}}[\text{CFTRi}]{\left[\text{PKAa}\right]}^{\beta }-k_{\text{CFTRr}}[\text{CFTRa}]$$

(5) $$F_{\text{ATP}}=k_{\text{ATPflux}}[\text{CFTRa}]$$

where the exponent β is used to modulate the relationship between PKA activation and CFTR activation. For simplicity it is assumed that *F*_ATP_, the release rate or flux of ATP (via pannexin 1, Sridharan et al., 2010), is proportional to activation of CFTR. In addition to Eqs 1–5, our model assumes conservation of GP, PKA, and CFTR:

(6) $$\begin{array}{l} \left[\text{GPtotal}\right]=\left[\text{GPi}\right]\left[\text{GPa}\right]\\ \left[\text{PKAtotal}\right]=\left[\text{PKAi}\right]\left[\text{PKAa}\right]\\ \left[\text{CFTRtotal}\right]=\left[\text{CFTRi}\right]\left[\text{CFTRa}\right] \end{array}$$

**Figure 2:**
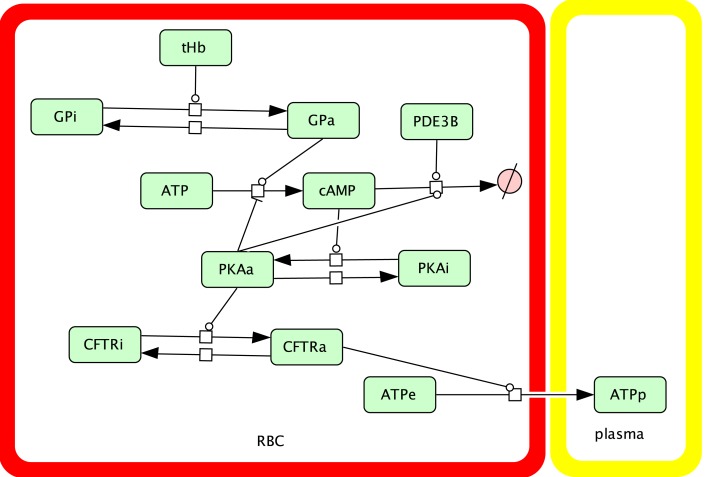
**Schematic showing the components and interactions included in our dynamic model of the oxygen-dependent erythrocyte ATP release pathway**.

Figure 2 shows the ATP release pathway model that was originally created using the free software package Cell Designer (http://celldesigner.org). Solution of Eqs 1–6 was implemented in Matlab (Mathworks, Natick, MA, USA) to allow more flexibility in exploring the model (e.g., specifying time-dependent saturation functions and automatically integrating and averaging results over time). A version of our Matlab simulation code is included online as Supplementary Material.

Parameters used in Eqs 2–3 were initially those used by Williamson et al. (2009) with modifications to reflect the much faster time-scale of erythrocyte ATP release (~100 ms vs. ~10 s in, Williamson et al., 2009) as reported for shear-induced ATP release in microfluidic experiments (Wan et al., 2008). It is important to recognize that available evidence suggests that shear stress applied to human erythrocytes activates the same pathway as does exposure to reduced O_2_ (Sprague and Ellsworth, 2012). Parameters for Eqs 1, 4, and 5 (in particular, the exponents α and β) were then set to represent those required for a similar time-scale, and were varied to match *in vitro* measurements of ATP release as a function of hemoglobin saturation (Sprague and Ellsworth, 2012) where erythrocytes were rapidly desaturated to various sO_2_ values starting from ~100% sO_2_. Although the dynamics of O_2_-dependent erythrocyte ATP release are important *in vivo*, no dynamic measurements are currently available (Sprague and Ellsworth, 2012). Therefore, we chose to use brief desaturation steps of fixed duration (40 ms) and varying magnitude (38–84%) to investigate the sO_2_ dependence of our dynamic ATP release model. The stimulation time of 40 ms was motivated by the experiments by Wan et al. (2008) in which changes in shear needed to be longer than ~6 ms (activation time) to produce changes in erythrocyte ATP release while the delay time between changes in shear and changes in ATP release was ~29 ms.

An underlying assumption in our model is that the O_2_-dependent pathway does not release ATP when hemoglobin is fully saturated with oxygen (i.e., AC_base_ = 0). Therefore, the sO_2_ dependence of the model was based on the results reported by Sprague and Ellsworth (2012) with the measured ATP release for 98% sO_2_ (5.4 nmol ATP per 4 × 10^8^ erythrocytes) taken to represent full saturation which was subtracted from the ATP release values for lower saturations. This yielded target ATP release values of 3.1, 7.1, and 13.1 nmol ATP/4 × 10^8^ erythrocytes for 61.8, 41.3, and 21.6% sO_2_, respectively. To enable the model to predict the inhibitory effect of insulin on O_2_-induced ATP release, as reported experimentally (Hanson et al., 2009, 2010), the value of PDE3_rel_ was adjusted to model erythrocyte ATP release upon exposure to reduced O_2_ tension in the presence of levels of insulin seen in pre-diabetes or required for the treatment of type 2 diabetes (Kanauchi et al., 2007; Ellis et al., 2010). Again, the ATP release values were adjusted for zero ATP release at full saturation, yielding a target value for ATP release of 11.8 nmol ATP/4 × 10^8^ erythrocytes at 15.7% sO_2_ without insulin and a target value of 2.77 at 20.9% sO_2_ with insulin, both determined experimentally (Hanson et al., 2009).

### Computational model of O_2_ and ATP transport in capillary networks

Numerical simulations of steady-state O_2_ transport were performed using an established time-dependent, finite-difference computational model (Goldman and Popel, 1999, 2000; Ellis et al., 2010; Sprague et al., 2010) that couples the continuum partial differential equations describing convective transport by flowing blood in the capillaries with equations describing O_2_ diffusion and consumption in the tissue. This model incorporates both dissolved and hemoglobin-bound O_2_ in the capillaries. Transport of O_2_ between the blood and tissue is described using a flux boundary condition with mass transfer coefficients calculated previously using a discrete erythrocyte model (Eggleton et al., 2000). In the model presented here, for all O_2_ transport simulations, a capillary network reconstructed from experimental data was used (Fraser et al., 2012) in conjunction with hemodynamic parameters (erythrocyte velocity and hematocrit) determined from *in vivo* measurements in the rat extensor digitorum longus (EDL) muscle. The capillary network was discretized into 208 cylindrical segments and the tissue domain surrounding the capillaries, which had dimensions of 84 × 169 × 342 μm, was discretized into 632,315 computational nodes. Average capillary entrance saturations (63%) and the tissue O_2_ consumption rate (1.5 × 10^−4^ ml O_2_/ml/s) were set based on previous experimental data (Ellis et al., 2002). The geometric and hemodynamic data used in the blood-tissue oxygen transport calculations, as well as the resulting steady-state values for capillary sO_2_, are included online as Supplementary Material.

Numerical simulations of steady-state ATP transport within the capillary network were performed using a modified form of our time-dependent finite-difference computational model for intravascular O_2_ transport (Goldman and Popel, 2000). Based on a previously described ATP transport model (Arciero et al., 2008), the following continuum partial differential equation was solved for plasma ATP concentration [ATP] using the geometric, hemodynamic, and sO_2_ data described above and an initial ATP concentration of zero:

$$\begin{array}{rcll} \left(1-H_{\text{T}}\right)\frac{\partial }{\partial t}\left[\text{ATP}\right] & =-u\left(1-H_{\text{D}}\right)\frac{\partial }{\partial z}\left[\text{ATP}\right] & & \\ & \;+H_{\text{T}}C_{\text{0}}\left(1-C_{\text{1}}S\right)-\frac{2}{R}k_{\text{d}}\left[\text{ATP}\right] & & \text{(7)} \end{array}$$

where *u* is the averaged cross-sectional blood velocity at any axial location *z*, *H*_D_ is the discharge hematocrit, *H*_T_ is the tube hematocrit, and *R* is capillary radius. As previously defined (Arciero et al., 2008), the constants *C*_0_ and *C*_1_ are used to produce a linear approximation to the (steady-state) ATP release rate as a function of oxyhemoglobin saturation *S*, while the constant *k_d_* approximates steady-state degradation of ATP by the endothelium (see Table 1 for parameter values). To model the effect of elevated plasma insulin on ATP release, we decreased C_0_ by 50% based on experimental measurements.

**Table 1:** **Parameters for ATP release pathway and ATP transport**.

Parameter	Value
AC_base_	0
*k*_cAMPf_	49.5
*k*_cAMPi_	2.47
*v*_0_	101
*K*_PDE3_	1
*k*_PKAf_	60.5
*k*_PKAr_	10.0
*k*_GPf_	25.0
α	1.2
*k*_GPr_	3.36
*k*_CFTRf_	181
β	6.3
*k*_CFTRr_	11.3
*k*_ATPflux_	2
GP_total_	1
PKA_total_	1
CFTR_total_	1
*C*_0_	1.4 × 10^−9^ mol/s·cm^3^
*C*_1_	0.891
*k*_d_	2.0 × 10^−4^ cm/s

In a previously reported model for microvascular regulation (Arciero et al., 2008), seven representative unbranched vessel segments (artery, large arteriole, small arteriole, capillary, small venule, large venule, vein) were included in the simulation and the inlet [ATP] in the farthest upstream vessel (artery) was set at 0.5 μM. This led to an inlet [ATP] in the capillary of approximately 0.25 μM. Therefore, we used this value for inlet [ATP] in our capillary network simulations. However, since this value depends on other modeling assumptions in the work of Arciero et al. (2008), we also considered the case where [ATP] is zero at the entrance of our capillary network to more clearly illustrate the contribution of erythrocyte-derived ATP in the capillary bed to plasma [ATP].

The spatial distribution of steady-state sO_2_ values was computed for the 3D capillary network based on experimental measurements of entrance sO_2_ and total erythrocyte supply rate in the network. The same steady-state sO_2_ distribution was used for both normal and impaired ATP release. To solve Eq. 7 for steady-state [ATP] once steady-state sO_2_ values had been calculated, an arbitrary initial condition ([ATP] = 0) was chosen and simulations were run until [ATP] became constant in all capillary segments. Although the present work focuses on steady-state capillary [ATP] distributions, our computational model is capable of simulating changes in intravascular [ATP] for time-varying blood flow, O_2_ consumption rate, or erythrocyte ATP release.

As noted above, to have a major impact on oxygen delivery to meet increased demand, the endothelial signal produced by ATP released in capillaries or venules must be conducted upstream and stimulate arteriolar dilation. Therefore, we integrated [ATP] obtained from Eq. 7 to estimate the total dilatory signal σ_dilation_ produced by ATP released from erythrocytes in the capillary network:

(8) $$\sigma_{\text{dilation}}=\overset{208}{\underset{i=1}{\sum }}{\left[\text{ATP}\right]}_{i}\exp\left(-\frac{L-z_{i}}{\lambda }\right)$$

where *L* is the arterio-venous length of the capillary network, and *z_i_* is the axial location of the segment with ATP concentration [ATP]_i_. The parameter λ determines the length scale of attenuation of the conducted signal and is set to 1 cm based on the work of Arciero et al. (2008) who obtained this approximate value from highly variable (0.15–1.6 cm) experimental data (Xia and Duling, 1995). Note that in the present work, σ_dilation_ is simply used as a measure of the dilatation signal originating in the capillaries. Since arterioles are not included in this model, we cannot use σ_dilation_ to change vascular diameters.

## Results

### O_2_-dependent ATP release pathway

**Figure 3:**
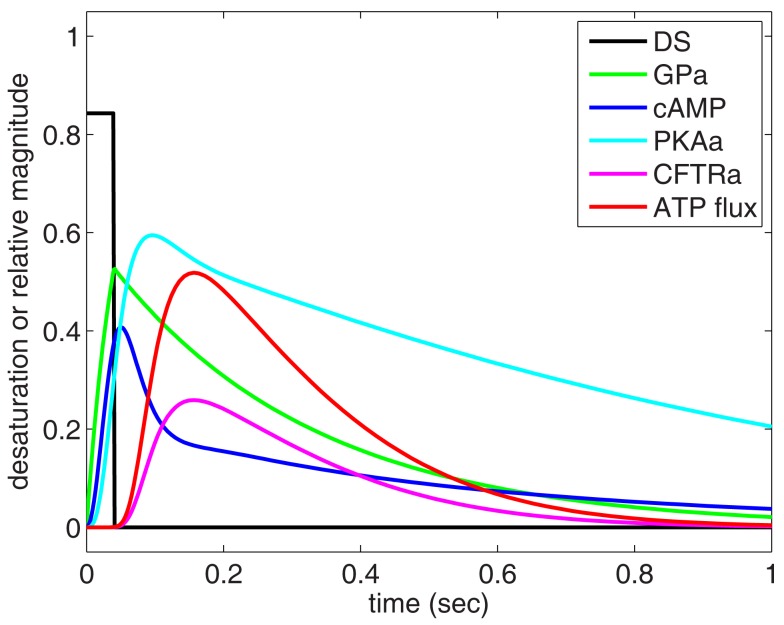
**Dynamic behavior of our model of oxygen-dependent erythrocyte ATP release**. Hemoglobin oxygen saturation is decreased from 100 to 15.7% for 40 ms, resulting in activation of the ATP release pathway with an initial delay in ATP release followed by a peak in ATP flux at approximately 150 ms. The pulse of hemoglobin desaturation results in a total release of ATP (area under ATP flux curve) that can be compared to experimental measurements. The ATP release delay and peak times are consistent with shear-dependent release dynamics measured by Wan et al. (2008), and the GP activation time-scale is consistent with the measurements of Hein et al. (2005).

Using Eqs 1–6 and parameters listed in Table 1, we simulated the response of the O_2_-dependent ATP release pathway in human erythrocytes to a 40 ms period of oxyhemoglobin desaturation starting from an initial condition with all variables equal to zero. Figure 3 shows the predicted dynamic response of this pathway to a step change in sO_2_ from 100 to 15.7% (i.e., increase in [tHb] from 0 to 0.843) with a duration of 40 ms. Figure 3 describes a temporal relationship among the components of the signaling pathway in which GP is activated first by hemoglobin desaturation with cAMP peaking at ~51 ms and the ATP release rate (or flux) peaking at ~157 ms after this physiological stimulus. Following the return to full hemoglobin saturation, ATP flux returns to zero in less than 1 s. Time-dependent results using this ATP release model will be useful when integrated into future dynamic models of microvascular flow regulation based on O_2_-dependent ATP release from erythrocytes.

To relate the results shown in Figure 3 to experimental measurements, cAMP and ATP flux are integrated over the time required for the desaturation step to turn on and off and for the release of ATP to stop. It is important to recognize that our model computes relative activation and ATP flux values for an average pathway without considering the number of these GP-coupled pathways present in an individual erythrocyte. Therefore, to compare our results directly to measurements of ATP release, we normalized our findings to agree with experimental measurements (Hanson et al., 2009) at 15.7% sO_2_ ([tHb] = 0.843).

**Figure 4:**
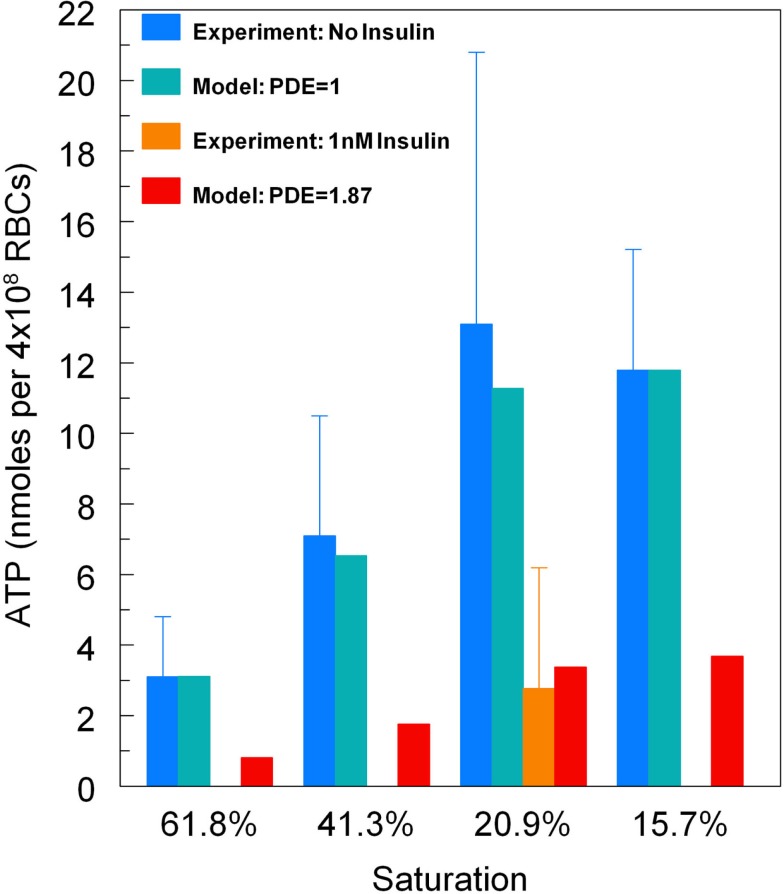
**Model predictions for total ATP release vs. hemoglobin saturation**. For baseline levels of PDE3 activity, predicted ATP release matches experimental measurements (Sprague and Ellsworth, 2012). For an 87% increase in PDE3 activity, ATP release decreases as seen for erythrocytes incubated in insulin (Hanson et al., 2009). Here ATP release has been normalized so that at 15.7% sO_2_the model matches the ATP release measurements without insulin.

**Figure 5:**
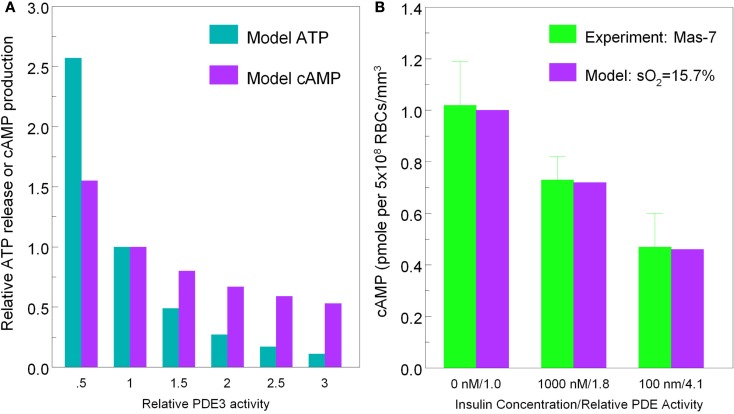
[**(A)**, left] Model predictions for ATP release (cyan bars) and cAMP production (purple bars) at 15.7% hemoglobin saturation vs. PDE3 activity. [**(B)**, right] Measured erythrocyte cAMP production (green bars) in response to Mas-7 stimulation vs. insulin concentration (0, 1 mM, 100 nM) and model predictions of cAMP production (purple bars) at 15.7% sO_2_ vs. PDE3 activity (PDE3_rel_ = 1, 1.8, 4.1).

To demonstrate that predictions from our model of the erythrocyte ATP release pathway agree with experimental data, in Figure 4 we plotted total ATP release vs. sO_2_ where the desaturation magnitude [tHb] = 1−sO_2_. This comparison confirms that our model captures the dependence of ATP release on sO_2_ under conditions in which PDE3_rel_ = 1. Importantly, when PDE3 activity is increased by 87% (PDE3_rel_ = 1.87) our model predictions of amounts of ATP released when erythrocytes are exposed to reduce O_2_ closely match ATP levels measured in the presence of 1 nM insulin (Hanson et al., 2009). The 87% increase in PDE3 activity is based on effects of insulin on the activity of this PDE in adipocytes (Kitamura et al., 1999). Thus, this model allows us to predict the inhibitory effect of insulin-induced increases in PDE3 activity on cAMP levels and ATP release from erythrocytes in which hemoglobin saturation is reduced to 15.7% (Figure 5A). As depicted in Figure 5B, the model also allows prediction of the level of PDE3 activity required to replicate experimental measurements of erythrocyte cAMP initiated by direct activation of Gi with mastoparin 7 (Mas-7) in the absence and presence of PDE3-stimulating concentrations of insulin (Hanson, 2009; Hanson et al., 2010).

### Coupled O_2_-ATP transport in capillary networks

**Figure 6:**
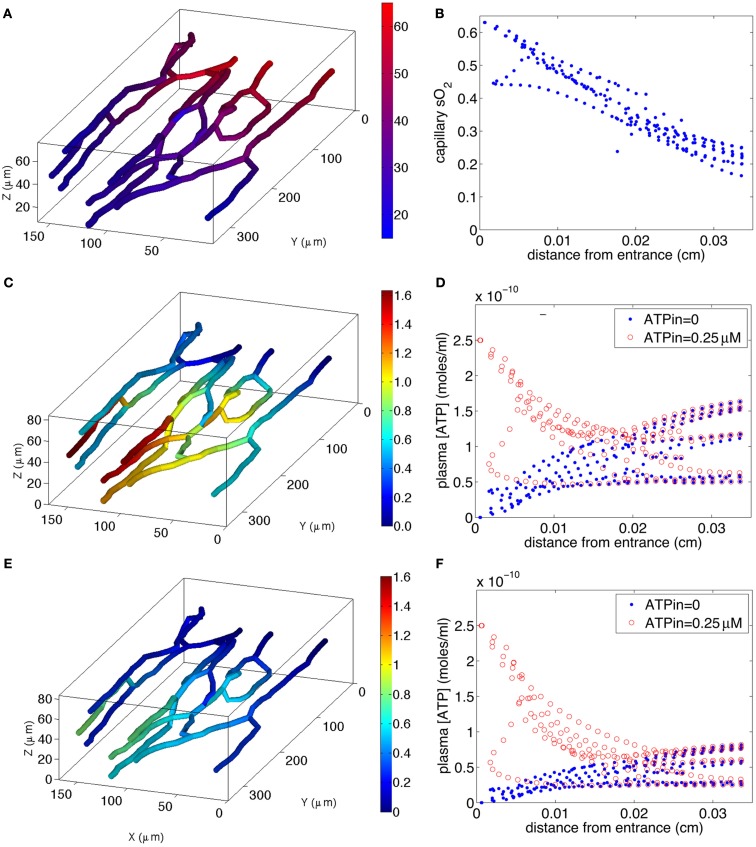
**Model of steady-state oxygen-dependent ATP distribution in capillary networks**. Top two panels: calculated capillary sO_2_ distribution [**(A)**, left] in 3D network and [**(B)**, right] as a function of distance *z* from capillary entrance. Middle two panels: calculated ATP distribution for normal ATP release [**(C)**, left) in 3D network with ATP_in_ = 0 and [**(D)**, right) as a function of *z* for ATP_in_ = 0 and ATP_in_ = 0.25 μM. Bottom two panels: calculated ATP distribution for impaired ATP release due to increased plasma insulin [**(E)**, left] in 3D network with ATP_in_ = 0 and [**(F)**, right) as a function of *z* for ATP_in_ = 0 and ATP_in_ = 0.25 μM. The color bar in (A) indicates the variations in sO_2_ (17–63%) in the 3D capillary network, while the color bars in **(C,E)** indicate the variations in [ATP] [0–1.6 μM in **(C)** and 0–0.8 μM in **(E)**] in the network.

The simulated O_2_ distribution in our reconstructed capillary network is shown in Figure 6A, and the 3D ATP distributions calculated for O_2_-dependent erythrocyte ATP release in the absence and presence of insulin are shown in Figures 6C,E, respectively. The O_2_ transport model shows a nearly linear decrease in sO_2_ (Figure 6B) with little variation among capillaries, except for one capillary with counter-current flow.

If the capillary inlet [ATP] (ATP_in_) is set to zero, all capillaries for normal ATP release (blue symbols in Figure 6D) show an increase in [ATP] although the variation among vessels is much greater than the variation in capillary sO_2_. The variation in [ATP] is due to the capillary network geometry and convective transport of ATP combined with the degradation of ATP by ecto-ATPases. When ATP release from erythrocytes is impaired (blue symbols in Figure 6F), the rate of increase in [ATP] with distance down the network is much less. The mean capillary ATP concentrations in the absence and presence of insulin are 0.076  and 0.038 μM, respectively, implying a 50% decrease when plasma insulin is increased to values found in pre-diabetes.

If ATP_in_ = 0.25 μM (Arciero et al., 2008), degradation of ATP results in a decrease in capillary [ATP] until approximately half way down the network for normal release (red symbols in Figure 6D), and slightly further for impaired release (red symbols in Figure 6F). In the case in which ATP release is impaired, [ATP] nearly plateaus suggesting that the rate of release is approximately equal to the rate of degradation. When ATP release is unimpaired, [ATP] either plateaus or increases substantially toward the venular end of the network. The mean ATP concentrations in the absence and presence of insulin are 0.115 and 0.077 μM, respectively, a 33% decrease in the presence of insulin. Thus, for either value of ATP_in_, there is a substantial decrease in capillary ATP when low O_2_-induced ATP release from erythrocytes is impaired.

Calculated values of σ_dilation_ show behavior similar to that of mean ATP, since the length scale of signal attenuation (λ = 1 cm) is much greater than the arterio-venous length (~350 μm) of the capillary network. For ATP_in_ = 0, σ_dilation_ is 1.6 × 10^−8^ and 0.8 × 10^−8^ for normal and impaired ATP release, respectively (i.e., a 50% decrease), while for ATP_in_ = 0.25 μM σ_dilation_ is 2.4 × 10^−8^ for normal vs. 1.6 × 10^−8^ for impaired release (i.e., a 33% decrease).

## Discussion

ATP release from erythrocytes in response to both physiological and pharmacological stimuli has been suggested to contribute to the regulation of perfusion distribution in skeletal muscle (Ellis et al., 2010; Sprague et al., 2010). Mechanical deformation (Wan et al., 2008) and exposure to reduced O_2_ tension (Ellsworth et al., 2009), both of which occur in small skeletal muscle microvessels, stimulate erythrocyte ATP release. Such a mechanism provides a means by which the distribution of perfusion can be regulated dynamically (Ellsworth, 2000, 2004; Ellsworth et al., 2009; Pittman, 2010). Extensive experimental evidence has established that ATP release from erythrocytes varies in response to changes in the levels of O_2_ tension to which these cells are exposed (Ellsworth et al., 1995; Jagger et al., 2001) and that increases in microvascular ATP concentrations result in vasodilation that is conducted to the feed arterioles, promoting an increase in oxygen supply to downstream tissues (McCullough et al., 1997; Collins et al., 1998; Ellsworth, 2000). Importantly, it has been reported that low O_2_-induced ATP release and subsequent vascular responses occur at a sufficiently fast time-scale (between 100 and 500 ms) to allow physiologically relevant dynamic regulation of O_2_ supply (Dietrich et al., 2000; Wan et al., 2008; Ellis et al., 2010). In addition, defects in O_2_-dependent ATP release by erythrocytes are present in both type 2 diabetes and pre-diabetes (Sprague et al., 2006, 2010, 2011) two disorders which are associated with peripheral vascular disease. Taken together, these results support a role for low O_2_-induced ATP release from erythrocytes in the microcirculation of skeletal muscle as a means by which the distribution of microvascular perfusion can be dynamically regulated (Ellsworth, 2000, 2004; Ellsworth et al., 2009; Pittman, 2010).

Under normal physiological conditions, ATP release would provide an effective mechanism by which perfusion could be dynamically regulated to meet tissue O_2_ needs. However, under conditions in which systemic or local microvascular hematocrit is significantly reduced, other mechanisms would be required to increase flow to the tissue to minimize tissue hypoxia (Roy et al., 2012). Such protective mechanisms would likely be the same as those which would become important under conditions in which low O_2_-induced ATP release from erythrocytes is defective as occurs in humans with pre-diabetes (high insulin levels) or type 2 diabetes.

In recent years, a detailed description of microvascular O_2_ transport has been developed based on a number of multiscale experimental and theoretical studies (Popel, 1989; Ellsworth et al., 1994;Pittman, 1995, 2005, 2011; Goldman, 2008). A number of studies have described convective and diffusive transport of O_2_ inside individual erythrocytes, in single capillary and arteriolar segments, and in arrays or networks of multiple interacting capillaries, arterioles, and venules. A full understanding of the contribution of erythrocyte-derived ATP to the regulation of blood flow distribution in the skeletal muscle microcirculation requires similar theoretical and experimental assessment of the release of ATP from erythrocytes, the diffusion and binding of ATP to the purinergic receptors on the vascular endothelium, and the impact of conducted vasodilation initiated in the microcirculation on flow in both individual vessels and complex vascular networks.

Here we present two experiment-based modeling components of our evolving multiscale approach to the characterization of the regulation of microvascular perfusion in response to low O_2_-induced ATP released form erythrocytes. Most importantly, we have a developed a novel dynamic model of the signaling pathway within the erythrocyte that is responsible for this ATP release. The ATP release model is based on previous experimental work describing the components of this pathway. The predictions of the model are consistent with reported time-scales for ATP release (Dietrich et al., 2000; Wan et al., 2008) and agree with measured ATP release from erythrocytes as a function of hemoglobin sO_2_ and hydrolysis of cAMP by PDE3. The model also predicts the time course of ATP release, which is a vital determinant of the effectiveness of microvascular flow regulation, and allows us to investigate how defects in that release would compromise optimal O_2_ delivery. Finally, the model can be used to predict whether corrections of defects in this pathway may be important therapeutic targets in the treatment of vascular dysfunction associated with diseases such as pre-diabetes and type 2 diabetes in humans.

Little is known about the mechanism linking reduced hemoglobin saturation and G-protein activation in the erythrocyte. However, the time course used in our model for erythrocyte ATP release is similar to the time course reported for other G-protein activated signaling pathways. For example, dopamine activation of a G-protein coupled potassium current (~250 ms from activation to increased potassium current) in the mouse midbrain (Ford et al., 2009) and α2_A_ adrenergic receptor activation of Gi (<100 ms) in HEK293 cells stimulated with norepinephrine (Hein et al., 2005) are within the time-scale modeled here for erythrocyte ATP release.

In addition to developing a model for low O_2_-induced ATP release from erythrocytes, we present a model of ATP transport in skeletal muscle capillary networks. This model incorporates a reconstructed 3D network and is based on *in vivo* measurements of rat skeletal muscle. This new approach permits the simulation of realistic capillary ATP transport and the consequences for the vasodilatory signal that is conducted from sites of increased O_2_ demand (i.e., the capillary bed) to augment blood flow. In addition, we have employed the model to enhance our understanding of the consequences of insulin-induced decreases in erythrocyte ATP release (measured *in vitro*) on conducted signaling and, consequently, on the regulation of local O_2_ delivery in a realistic skeletal muscle capillary network. The computational model suggests that details of capillary network geometry and hemodynamics are important in determining the manner in which ATP signaling from erythrocytes is conducted upstream to regulate microvascular O_2_ delivery. Importantly, the information obtained from this capillary network model will be important for the further development of a full multiscale description of the regulation of microvascular O_2_ delivery in skeletal muscle, allowing us to connect local tissue function (oxygen tension and consumption; Fraser et al., 2012) with mechanisms involved in the regulation of O_2_ supply.

### Model assumptions and limitations

We modeled the O_2_-dependent ATP release pathway of the erythrocyte with a series of five biochemical steps described by four time-dependent ordinary differential equations. Although this model can be solved very rapidly using Matlab on a personal computer, it did require inclusion of a number of parameters (~15 rate constants and half-maximum concentrations) that have not been directly measured. This model also requires assumptions about how oxyhemoglobin desaturation activates Gi and how CFTR activation opens the pannexin 1 channel leading to ATP release. Although most of the biochemical steps used are understood qualitatively and most of the constants can be estimated from measurements of ATP release and cAMP production, one could question the need for such a complex model. If the objective had been only to describe existing data, then a phenomenological model might have been adequate. However, since we were seeking a model that could predict the detailed dynamics of ATP release and the impact of specific changes in the pathway (e.g., increased PDE3 activity), the complexity of the present model was necessary.

A primary assumption made in constructing our model of intravascular ATP transport was that ATP released from erythrocytes into the surrounding plasma becomes well-mixed across the vessel lumen, such that the mean plasma ATP concentration determines ATP binding to endothelial P_2Y_ receptors. Although this may be correct, an alternative possibility is that ATP released near the vessel wall is more important than the mean ATP concentration. If so, this might alter ATP action within the microvasculature as well as other characteristics of the flow regulation system. Presently, it is not known how ATP is radially distributed within the vascular lumen.

There are several limitations to the present model in terms of predicting ATP release by erythrocytes under conditions other than those described here. First, since most parameters have not been directly measured, their values are approximate and may need to be revised once more detailed time-dependent data becomes available. Second, since the relevant details of Gi activation and pannexin 1 opening are not currently known, these were treated phenomenologically. Third, our model has ignored other known mechanisms inducing erythrocyte ATP release including both shear-dependent release, which appears to occur through the same pathway, and receptor-mediated release which utilizes a distinct signaling pathway (Ellsworth and Sprague, 2012). Expansion of this model to include these components would enhance the robustness and utility of the current model and should enable one to predict the total time-dependent release of ATP by an erythrocyte that would occur *in vivo* under a wide range of physiological or pathophysiological conditions.

### Model testing

As described previously (Ellsworth et al., 2009), testing our full multiscale model of blood-tissue oxygen transport and its regulation based on ATP release by erythrocytes requires *in vivo* experiments. However, a large amount of development and testing of individual model components will be required prior to performing direct comparisons of model predictions to *in vivo* behavior. Currently available microfluidic devices, similar to those of Wan et al. (2008), will allow measurements of the dynamics of O_2_-dependent erythrocyte ATP release enabling us to experimentally test the ATP release model’s predictions under a wide range of conditions (e.g., increased insulin). These results will provide important dynamic information for the refinement of the model setting the stage for further experimental testing.

### Model predictions and novel experiments

The ATP pathway model we have developed allows us to make predictions about how the various parameters in Eqs 1–6 interact to determine ATP release, and these predictions can be used as the basis for novel experiments. For example, in humans with type 2 diabetes it is known that there is an approximate 40% decrease in expression of Gi protein in the erythrocyte membrane (Sprague et al., 2006). If we implement this in our model by decreasing GP total accordingly in Eq. 6, we would predict a decrease in ATP release of approximately 60% when sO_2_ is decreased briefly from 100 to 15.7%. This prediction links a decrease in a known component of the erythrocyte ATP release pathway with a known measured outcome, i.e., reduced low O_2_-dependent ATP release from erythrocytes of humans with type 2 diabetes. An attempt to remedy this defect could be simulated in the model by decreasing PDE3 activity as done experimentally with the selective PDE3 inhibitor cilostazol. Under these conditions, a 60% decrease in PDE3 activity (PDE3rel = 0.6) would return O_2_-dependent ATP release back to its normal level. Thus, the model presented here enables us to predict the effect of a known defect in the release pathway on ATP release, and then provides us with a mechanism to evaluate how this defect could be most effectively remedied. This in turn motivates new *in vitro* and *in vivo* experiments to test these predictions in erythrocytes and in intact muscle, to determine if this approach has potential for treating humans with type 2 diabetes.

## Conclusion

The architectural, biophysical, and temporal complexity of microvascular O_2_ transport makes complete understanding of its regulation difficult. However, it is clear that a full understanding of the regulation of microvascular O_2_ delivery requires a detailed multiscale approach. The novel theoretical models described here for low O_2_-induced ATP release from erythrocytes at the intra-erythrocyte and capillary network levels form the basis for our dynamic systems biology model of microvascular blood flow regulation.

## Conflict of Interest Statement

The authors declare that the research was conducted in the absence of any commercial or financial relationships that could be construed as a potential conflict of interest.

## Supplementary Material

The Supplementary Material for this article can be found online at http://www.frontiersin.org/Computational_Physiology_and_Medicine/10.3389/fphys.2012.00246/abstract
